# Medical and Surgical Memoirs

**Published:** 1876-09

**Authors:** 


Medical and Surgical Memoirs, containing Investigations on the Geographical Distribution, Causes, Nature, Relations, and Treatment of Various Diseases. 1855-1876. By Joseph Jones, M. D., Professor of Chemistry and Clinical Medicine, Medical Department, University of Louisiana, Visiting Physician of Charity Hospital, formerly Surgeon in the Provisional Army of the Confederate States, etc., etc. Volume 1, 8 vo., pp. 883. Containing Introduction to the Study of Diseases of the Nervous System, Investigations on Traumatic Tetanus, Epilepsy, Paralysis, and Cerebro-Spinal Meningitis; Clinical Observations on Diseases of the Lymphatic and Circulatory Systems, and of the

Liver and Kidneys; Investigations and Researches on Pneumonia, and Observations on Diseases of the Osseous System, with illustrations of 800 cases of Disease, 400 physiological experiments, 95 analyses of the blood and urine, and 60 tables illustrating the symptoms and mortality of diseases under different modes of treatment and in different climates. The book is furnished bound in cloth and leather at $5.00, the postage being 50 cents, and will be sent by mail to any part of the United States, Canada, or England, upon the receipt of $5.50, in post-office money order, or by express, by addressing Joseph Jones, M.D., box 1500, P. O. New Orleans, Louisiana.

While we have not had the opportunity to examine this work with the care which our intimate knowledge of the author and his eminently deserving labors invited, we are sufficiently impressed with its merits to commend it without hesitation to every medical man who feels the least interest in the literature of his profession, or who desires to keep pace with the progress of scientific investigations, as in both particulars Dr. Jones deserves to be recognized as a distinguished authority, and as well deserving the reputation of being one of the most reliable, patient, and laborious workers in the United States, and that, too, under circumstances to which many of our best men in the Southern States have succumbed. Besides, we think that Southern medical men particularly should exhibit a special interest in sustaining Prof. Jones in a work which is not only creditable to himself, but to the whole profession of the South, from which section we have had little or no contributions to medical science in any permanent form. Besides, the very fidelity and exhaustiveness of research, presenting such a marked feature of this book, has made it uninviting to publishers, and thus the entire burden and risk of its publication has been thrown upon its author, and we should see to it that he should not only not suffer loss, but that the great labor and ability expended in its preparation should be amply rewarded.

In conclusion of this very imperfect notice, we are pleased to put upon record the following high testimonial from the eminent editor of the *Sanitarian* to the value of Prof. Jones' labors. He says: "The least we can ask our readers and all students of physiology and etiology to do, is to avail themselves of the benefits herein comprehended by placing this and each succeeding volume of Prof. Jones' series of Medical Memoirs in their own libraries. The recognition of Prof. Jones'

former works, as pre-eminently original and important, by such distinguished physicians as Flint and Milne Edwards, and his contributions to the U. S. Sanitary Commission's publications, on the Causes of Mortality in the Military Prisons of the South, is the best of all authority upon the subject, are abundant guarantee of our author's single-hearted purpose, and the success with which he prosecutes the objects of his research." J. P. L.

